# Experimental Research on Quarry Wastewater Purification Using Flocculation Process

**DOI:** 10.3390/molecules30132761

**Published:** 2025-06-26

**Authors:** Yongjie Bu, Kangjian Zeng, Heng Yang, Aihui Sun, Qingjun Guan, Shuang Zhou, Wenqing Peng, Weijun Wang, Peng Ge, Yue Yang

**Affiliations:** 1 School of Resource Environment and Safety Engineering, Hunan University of Science and Technology, Xiangtan 411201, Chinapengwenqing@163.com (W.P.); wjwang@hnust.edu.cn (W.W.); 2 Hunan Province Key Laboratory of Coal Resources Clean-Utilization and Mine Environment Protection, Hunan University of Science and Technology, Xiangtan 411201, China; 3 School of Minerals Processing and Bioengineering, Central South University, Changsha 410083, China

**Keywords:** quarry, wastewater treatment, flocculant, DLVO

## Abstract

The flocculation-based purification of quarry wastewater continues to pose a significant challenge in mineral processing and environmental engineering, primarily due to persistent turbidity issues and inefficient floc settling behaviour. In this study, we systematically investigate the synergistic effects of organic and inorganic flocculants to reduce turbidity and improve floc settling performance. Through a series of optimised experiments using polyaluminium chloride as an inorganic flocculant, polyacrylamide as an organic flocculant, and calcium oxide as a pH regulator agent, the treatment efficiency was evaluated. Under the optimal conditions with 200 g/m^3^ CaO as the regulator agent and 2.5 g/m^3^ PAC and 12 g/m^3^ PAM as flocculants, the residual turbidity was reduced to 97.30 NTU, meeting stringent industrial discharge standards and enabling zero-discharge water reuse. Zeta potential measurements, optical microscopy, and DLVO theory collectively elucidated the interfacial interactions between flocculants and mineral particles, with zeta potential revealing electrostatic effects, microscopy visualising aggregation patterns, and DLVO theory modelling revealing colloidal stability, thereby mechanistically explaining the enhanced aggregation behaviour.

## 1. Introduction

Sand and gravel aggregates, as primary mineral commodities extracted through quarrying operations, have become fundamental construction materials for civil engineering applications. These granular resources are critically important in infrastructure systems spanning architectural structures, transportation networks, bridge engineering, hydraulic engineering, and hydroelectric facilities. Their high consumption volumes and technical irreplaceability substantially contribute to national infrastructure development and macroeconomic progression through three distinct mechanisms: serving as essential constituents in concrete matrices and pavement foundations; acting as economic catalysts through value chain industrial linkages; and enabling sustainable construction practices via localised material sourcing [1,2,3,4].

Quarrying and mineral processing operations generate substantial volumes of wastewater, which pose significant risks of water resource contamination in adjacent environments [5]. This effluent typically contains high concentrations of fine particulate matter, residual reagents, and other processing chemicals. Residual chemicals on the surface of sand and gravel will greatly affect the performance of concrete. If discharged without adequate treatment, these contaminants can severely degrade water quality, leading to ecological disturbances and long-term environmental impacts [6,7].

The progressive escalation of quarry effluent discharge volumes has highlighted critical deficiencies in conventional treatment systems, with current technological limitations becoming increasingly pronounced under elevated hydraulic loading conditions [8,9]. Post-treatment effluent contains elevated concentrations of suspended particulates, persistent residual processing reagents, and excessive turbidity levels. Untreated or inadequately processed discharges induce irreversible ecological degradation in both aquatic and terrestrial matrices, adversely impacting hydrological systems within mining precincts and compromising environmental integrity in adjacent residential zones [10].

Quarry wastewater treatment employs methodologies analogous to conventional wastewater management systems, primarily utilising physical separation, chemical precipitation, and biological degradation processes for contaminant removal. The general absence of heavy metal contaminants in such effluent streams enables comparatively simplified treatment configurations, with operational complexity being substantially reduced relative to metalliferous mine drainage systems [10,11,12].

Conventional quarry wastewater treatment methodologies typically encompass flocculent sedimentation, pH adjustment, oxidative chemical treatment, coagulant-based precipitation, and engineered wetland systems [8,13,14].

The application of the flocculation and sedimentation method in wastewater treatment is extensive and effective. It is used to purify water by adding coagulants to the sewage so that the suspended matter, colloidal impurities, and microorganisms in the water coalesce into larger flocs, which are then separated from the water using sedimentation or air flotation [15]. The settling speed of this wastewater treatment is fast, and the operation is simple; it is easier than dealing with secondary pollution; however, choosing the right flocculant is difficult [16].

Flocculent sedimentation processes are widely utilised in wastewater remediation, demonstrating proven efficacy in contaminant sequestration [17,18]. This process facilitates aqueous purification through the introduction of polymeric coagulants that induce the agglomeration of suspended solids, colloidal particulates, and microbial biomass into settleable floc structures, subsequently separated via gravitational settling or dissolved air flotation separation [19,20]. While demonstrating rapid sedimentation kinetics and operational simplicity, this methodology presents challenges including residual coagulant retention in treated effluents and necessitates the formulation optimisation of flocculants across diverse application scenarios.

Current flocculent sedimentation systems in mining operations predominantly utilise either inorganic or organic flocculants as standalone agents, neither of which can simultaneously achieve rapid sedimentation kinetics and optimal turbidity reduction [21,22]. Inorganic coagulants demonstrate rapid colloidal destabilisation kinetics through charge neutralisation mechanisms, effectively forming macroscopic floc structures with suspended particulates for swift water clarification. Conversely, organic polymeric flocculants exhibit enhanced particle agglomeration efficiency via bridging mechanisms, generating denser floc matrices with improved settling characteristics. Composite formulations integrating inorganic coagulants with organic polymers leverage synergistic interactions, enabling the concurrent optimisation of clarification efficiency and sedimentation velocity [23,24]. This dual-component approach not only enhances process economics through reduced chemical consumption but also minimises environmental footprints by decreasing residual sludge volumes, representing the strategic advancement of sustainable water treatment methodologies [25].

This study systematically evaluated the flocculation performance of inorganic, organic, and composite flocculants in quarry tailing wastewater treatment, with a focus on sedimentation kinetics and turbidity reduction efficiency. Experimental analyses incorporated zeta potential measurements, optical microscopy characterisation, and DLVO theory to elucidate the underlying mechanisms governing colloidal destabilisation and particle aggregation. The objective of this study is to provide a method to accelerate the settling rate and improve the water clarification capacity for quarry wastewater treatment.

## 2. Materials and Methods

### 2.1. Materials

**Figure 1:**
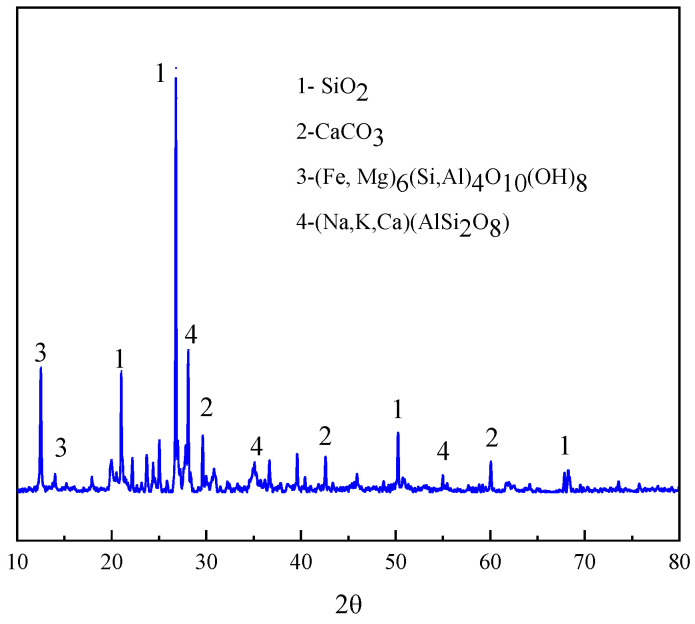
XRD diagram of mineral particles.

In this study, the slurry from a quarry beneficiation plant in Huzhou, Zhejiang Province, China, with a solid concentration of about 5.6 g/L, was used as a sample to accurately analyse the crystal structure of the mineral particles using X-ray diffraction (XRD, Bruker D6 PHASER, Billerica, MA, USA) technology, supplemented with X-ray fluorescence spectroscopy (XRF, Bruker S8 TIGER) technology, to quantify the elemental composition and content of the minerals, thereby revealing the chemical properties and composition of the mineral particles. The chemical composition of the mineral particles is presented in Table 1, while the XRD pattern is presented in Figure 1.

As evidenced in Table 1, silicon (Si) and aluminium (Al) constituted the predominant elements in the ore samples, accounting for 28.212% and 10.723%, respectively, whereas trace elements such as zinc (Zn) and gallium (Ga) were nearly undetectable.

The XRD analysis (Figure 1) reveals that quartz (SiO_2_) and feldspars ((Na, K, Ca)(AlSi_2_O_8_)), including orthoclase and albite, serve as the primary mineral phases. Additionally, calcite (CaCO_3_) and magnetite (Fe_3_O_4_) were identified, with the latter imparting magnetic properties to the ore.

**Figure 2:**
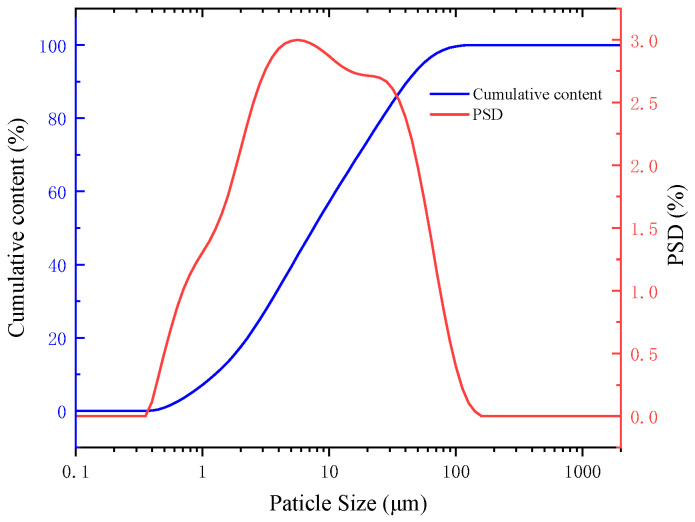
Mineral particle size and cumulative particle size distribution.

Mineral particle size is important for sedimentation and flocculation. A laser particle size analyser was used to analyse the distribution of mineral particles in the slurry. Figure 2 shows the mineral particle size distribution curve and the mineral particle cumulative distribution curve.

It is evident from the particle size distribution curve of the mineral particles that the particle size of the mineral particles in the quarry wastewater was mainly in the range of 1–100 μm, which indicates that the particle size in the quarry wastewater was small. It is observed from the cumulative distribution curve of mineral particles that the specific surface area of the mineral particles in the wastewater is 1.77 m^2^/g, d10 is 1.26 μm, d50 is 7.52 μm, and d90 is 41.37 μm. The particle size of the mineral particles is relatively fine, with 50% of the particles having a size smaller than 7.52 μm, and nearly 90% of the particles have a size smaller than 400 mesh.

The flocculation study employed two inorganic flocculants (purchased from Sinopharm Chemical Reagent Co., Ltd., Shanghai, China) and one organic flocculant (obtained from Shanghai Macklin Biochemical Technology Co., Ltd., Shanghai, China), including their combinations, to systematically evaluate their effects on quarry wastewater treatment.

### 2.2. Methods

#### 2.2.1. Flocculation Experiment

**Figure 3:**
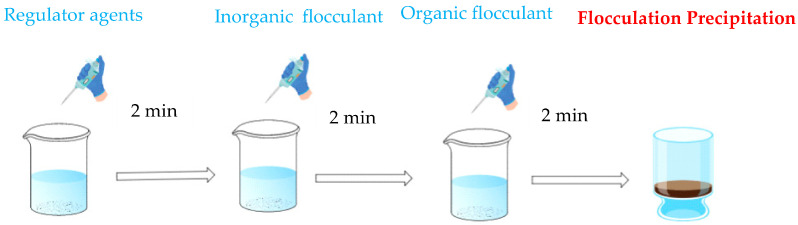
Flow chart of flocculation experiment.

At room temperature, 500 mL of wastewater was transferred into a 500 mL beaker; the regulator agents, inorganic flocculant, and organic flocculant were added in order and agitated for 2 min at a stirrer speed of 500 r/min. The wastewater was then transferred into a 100 mm height measuring cylinder. The “Height” in figures refers to the supernatant height thickness after flocculation. A suspension layer was observed in the wastewater settlement process, and the settling height and time were recorded. The upper layer of the clear solution was then tested for turbidity. All reagents were subjected to the above steps. Shown in Figure 3.

#### 2.2.2. Zeta Potential

The theory of zeta potential analysis is crucial for understanding the stability and interactions of tiny particles in a suspension system [26]. The magnitude of the absolute value of the zeta potential affects the stability between particles; the greater the absolute value of the zeta potential, the more stable the particles are and the less likely they are to settle. The significance of this theory is that it can help us to predict and control the aggregation behaviour of particles and optimise the flocculation process [27].

The charge on the particle surface affects the flocculation process; thus, zeta potential measurement is an important reference for investigating the flocculation process of fine particles. In this study, the electrophoretic method was used to determine the zeta potential values. The mineral particles were ground to a particle size of 5 μm, and 50 mg of this powder was taken in a 100 mL beaker each time, and 50 mL of deionised water was added and stirred with a magnetic stirrer for 5 min; the different types of flocculants were added, and the pH of the suspension was adjusted with HCl or NaOH and then stirred for 5 min; then, the supernatant layer was collected after one hour’s rest, and the zeta potential of the flocculant layer was measured using a zeta potential tester. In this study, the NanoZS90 instrument (Malvern Panalytical, Malvern, UK) was used for the determination of zeta potential on the surface of mineral microfine particles.

#### 2.2.3. Optical Microscope 

Optical microscopy provides a direct and clear method to analyse and observe the aggregation behaviour and structural changes in particles during flocculation [28,29]. The morphology, size, and specific distribution of flocs can be observed through optical microscopy to evaluate and analyse the flocculating effect of flocculants and optimise the flocculation conditions, and to observe the stability and settling characteristics of flocs after settling, which can be used to improve the water treatment and solid separation processes.

A 500 mL volume of wastewater is added to a 500 mL beaker and stirred at 500 rpm for 2 min at room temperature. The specific agent is added and mixed thoroughly; then, the suspension is poured into a 500 mL measuring cylinder and allowed to stand until no further changes occur. A drop of the upper clear liquid is taken from the burette and placed on a slide, to which a drop of 95% ethanol solution is added to evaporate water, and the slide is observed under a 640× light microscope.

#### 2.2.4. DLVO Theory

DLVO theory is a landmark achievement in the field of colloidal systems. DLVO theory provides a theoretical framework for understanding and predicting the stability of colloidal systems through the systematic consideration of van der Waals forces and the double-electron layer, which are the two interacting forces that play a dominant role among colloidal particles [30,31,32].

The size, shape, and surface properties of the particles were determined; then, the zeta potential of the particles was measured along with the ionic strength, pH, and temperature of the solution. Van der Waals forces and double-electron layer repulsion were calculated using the formulae. Combining the van der Waals forces of the double-electron layer repulsion yields the total potential energy of the particle, which in turn allows the stability of the particle to be analysed.

## 3. Results and Discussion

### 3.1. Wastewater Flocculation Experiment

#### 3.1.1. Experiments with Single Inorganic Flocculant

**Figure 4:**
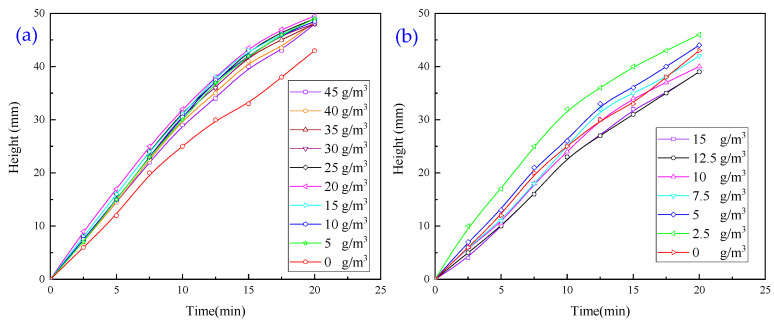
(**a**) Effect of PAC dosage on flocculation and sedimentation behaviour of wastewater. (**b**) Effect of PFS dosage on flocculation and sedimentation behaviour of wastewater.

Experiments with a single inorganic flocculant were carried out to investigate the effect of two inorganic flocculants on the flocculation and settling of wastewater, and the results are presented in Figure 4.

The experimental results showed that the addition of PAC alone did not significantly improve the settling and flocculation efficiency of solid particles. In particular, when the dosage of PAC exceeded a certain threshold, the settling effect decreased rather than increased.

Using PFS alone cannot significantly improve the settling and flocculation efficiency of solid particles, nor can it effectively achieve the rapid separation of mineral particles in wastewater.

#### 3.1.2. Experiments with Single Organic Flocculant

**Figure 5:**
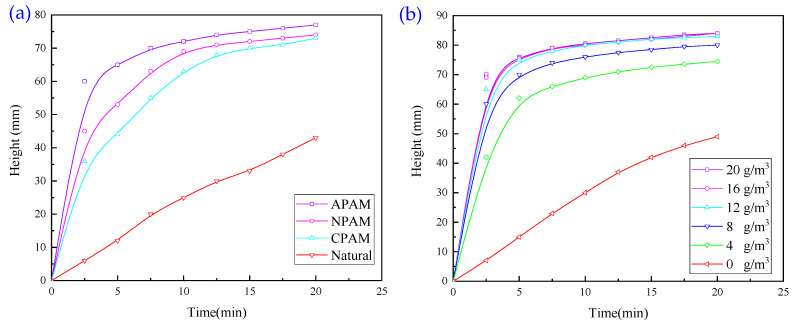
(**a**) Effect of PAM type on flocculation and sedimentation behaviour of wastewater. (**b**) Effect of PAM dosage on flocculation and settling behaviour of wastewater.

In order to investigate the effect of two organic flocculants on the flocculation and settlement of wastewater, experiments with a single organic flocculant were carried out, and the results are presented in Figure 5.

It is evident from Figure 5a that the settling effect was obviously better after adding 16 g/m^3^ of PAM. The overall effect of the polyacrylamide agent exhibits the following order: anionic type > nonionic type > cationic type. Therefore, the organic flocculant polyacrylamide agent used in this study is anionic PAM.

It is evident from Figure 5b that, after adding the organic flocculant PAM, the effect of wastewater sedimentation was significantly better; the sedimentation rate first increased with the dosage of the agent and then decreased slightly. When the dosage of the agent was 16 g/m^3^ and 20 g/m^3^, the rate of sedimentation was the largest and almost equal. Furthermore, sedimentation was reduced with an overdose of PAM. This is because excessive PAM interfered with the surface electrical properties of mineral particles, resulting in the generation of repulsive forces between particles, thus weakening the flocculation effect. In addition, the excessive use of chemicals not only caused a waste of resources but also increased the burden of residual chemicals in the subsequent treatment.

#### 3.1.3. Experiments with Single Regulator Agent

**Figure 6:**
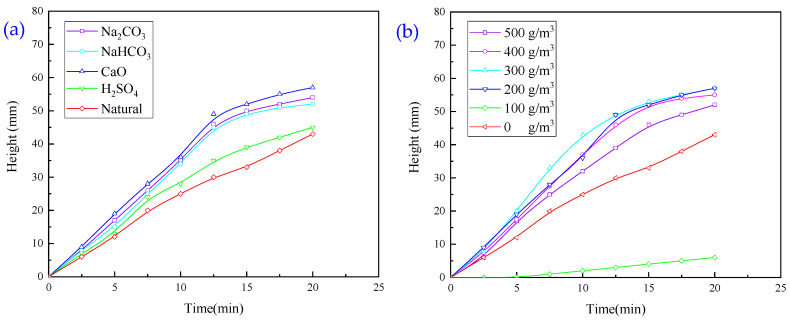
(**a**) Effect of regulator type on flocculation and settling behaviour of wastewater. (**b**) Effect of regulator dosage on flocculation and settling behaviour of wastewater.

The effect of regulator agents such as H_2_SO_4_, Na_2_CO_3_, CaO, and NaHCO_3_ on the flocculation and sedimentation behaviour of wastewater was investigated. H_2_SO_4_ was prepared as a 1 mol/L concentration solution with a dosage of 2 mL, and the rest of the solid powder was added by mass, and the dosages of CaO, Na_2_CO_3_, and NaHCO_3_ were 200, 378, and 600 g/m^3^, respectively, and the results are presented in Figure 6.

It is evident from Figure 6a that the addition of all four regulator agents accelerated flocculation and sedimentation; overall, the alkaline regulator agent exhibited a higher sedimentation rate than the acidic regulator agent H_2_SO_3_, and CaO had a greater sedimentation rate than Na_2_CO_3_, and NaHCO_3_ showed the best sedimentation effect. It is evident from Figure 6b that the addition of CaO improves the settling velocity to a certain extent, but it is still slower compared to that of PAC and PAM.

For a CaO dosage of 100 g/m^3^, the settlement speed is the smallest, and the turbidity of the solution is 617.26 NTU. The low settlement speed may be due to the presence of residual agents. When the CaO dosage was increased from 100 g/m^3^ to 500 g/m^3^, the settlement speed first showed an increase and then a small decrease in the super potential.

After adding a certain amount of CaO in the test process, the solution became clear. The turbidity value of the solution after the addition of 200 g/m^3^ and 300 g/m^3^ of CaO was 116.32 NTU and 101.91 NTU, respectively, which was a good indicator. Therefore, considering the test effect and economy, CaO was selected as the pH-regulating agent.

Considering the needs of the site and the cost, combined with the experimental effect, and choosing CaO as the pH regulator(Table 2), the experiments were conducted using a starting dosage of CaO of 200 g/m^3^ to obtain better results.

#### 3.1.4. Experiments with Flocculation Conditions Using a Combination of Two Agents

Due to the general effect observed in the single-agent test, the synergistic effects of different types of agents were investigated, i.e., the flocculation and sedimentation effects of the combination of PAM, PAC, and CaO.

**Figure 7:**
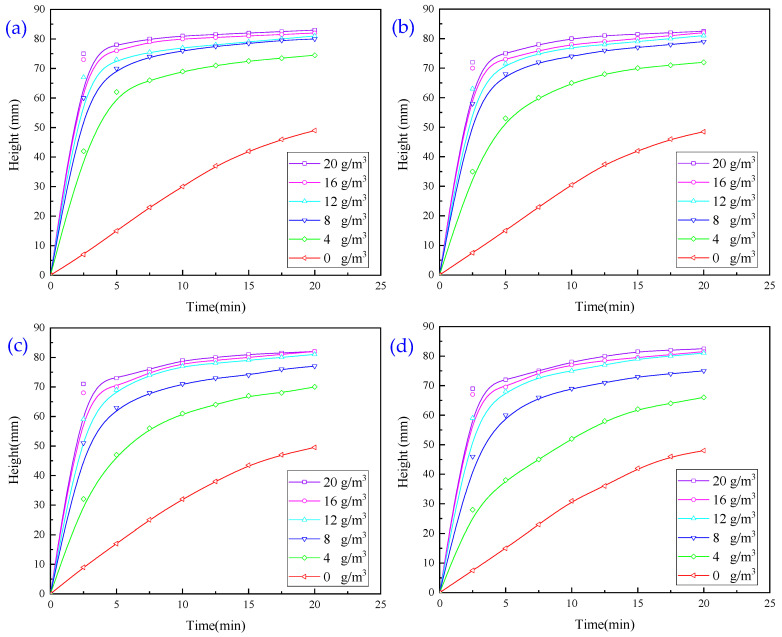
Effect of PAC on flocculation and sedimentation behaviour of wastewater: (**a**) PAC dosage of 5 g/m^3^; (**b**) PAC dosage of 10 g/m^3^; (**c**) PAC dosage of 20 g/m^3^; (**d**) PAC dosage of 30 g/m^3^.

PAC and PAM combination test: the effect of the PAC and 14 million influence anionic PAM combination was assessed using a pharmaceutical flocculation test, and the results are presented in Figure 7.

It is evident from Figure 7 that the combination of the two agents has a faster settling rate than PAC. Under different PAC dosages, the settling speed with a PAM dosage of 16 g/m^3^ is better than that with 12 g/m^3^; therefore, in the subsequent test, the PAC dosage is 5 g/m^3^, and the PAM dosage is 16 g/m^3^.

**Figure 8:**
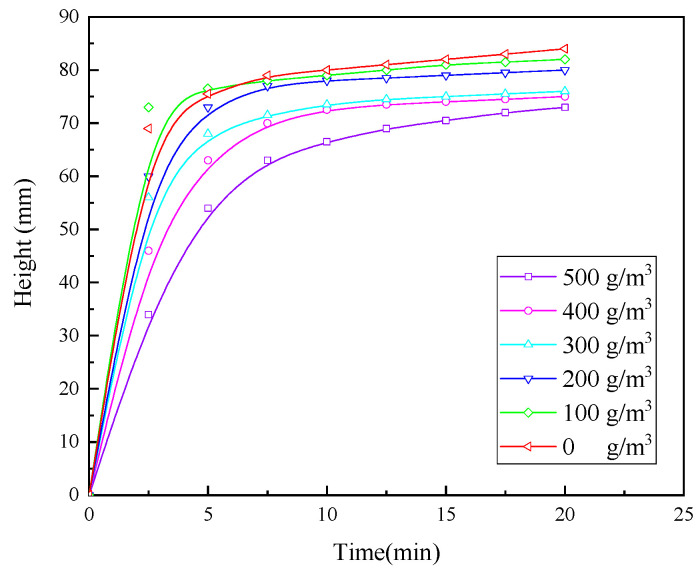
Effect of CaO dosage on flocculation and settlement behaviour of wastewater.

CaO and PAM combination test: with a 14 million anionic PAM dosage of 16 g/m^3^, the effect of CaO dosage on the flocculation and settlement behaviour of wastewater was investigated, and the results are presented in Figure 8.

It is evident from Figure 8 that, with a settlement time of 5 min or less and a CaO dosage of 100 g/m^3^, the settlement speed is the fastest; at this time, the combination of CaO and PAM can achieve a clearer supernatant, but the wastewater turbidity is 317.54 NTU, which does not meet the requirements of industrial discharge.

#### 3.1.5. Experiments with Flocculation Conditions Using a Combination of Three Agents

**Figure 9:**
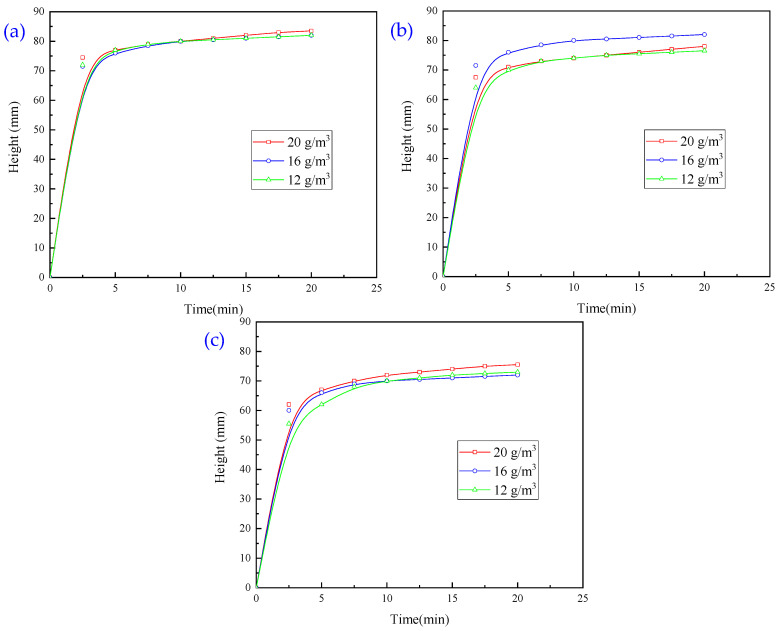
Effect of PAM dosage on flocculation and sedimentation behaviour of wastewater with (**a**) CaO dosage of 100 g/m^3^; (**b**) CaO dosage of 200 g/m^3^; (**c**) CaO dosage of 300 g/m^3^.

As the use of a single agent and the combined use of two agents did not achieve the desired results, we combined three flocculants, i.e., CaO, PAC and PAM, to investigate the effect of the combination on the settlement and flocculation of wastewater. In this experiment, a PAC dosage of 5 g/m^3^ and CaO additions of 100, 200, and 300 g/m^3^ were used. We studied the effect of PAM on the settlement height, and the results are presented in Figure 9.

It is evident from Figure 9 that the larger the amount of PAM added in 5 min, the higher the settling height and the faster the settling speed; the best settling effect is achieved when the amount of CaO is 200 g/m^3^ and the amount of PAM is 16 g/m^3^. The best pharmaceutical conditions are as follows: CaO 200 g/m^3^, PAC 5 g/m^3^, and PAM 16 g/m^3^. With these conditions, the turbidity of the upper layer of wastewater was 305.23 NTU, which did not meet the industrial discharge standards.

#### 3.1.6. Experiments with Optimised Conditions of Flocculating Agents

**Figure 10:**
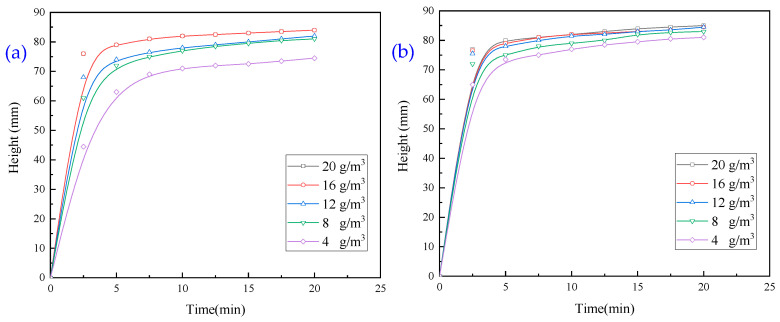
(**a**) Effect of PAM dosage on flocculation and settlement behaviour of wastewater. (**b**) Effect of PAM-New dosage on flocculation and settlement behaviour of wastewater.

The surface modification of PAM was performed, and the modified PAM (PAM-New) has small molecular groups on its surface, with stronger electrical and hydrophobic properties. The dosage of PAM was 5 g/m^3^, and that of CaO was 200 g/m^3^. The effect of PAM-New on the sedimentation and flocculation of wastewater was assessed, and the results are presented in Figure 10.

It is evident from Figure 10a that both the PAM settling speeds are faster, and in about 5 min, the settlement process is complete; however, the settling speed of PAM-New is slightly faster than that of PAM-mineral, and the turbidity of the upper layer of the liquid is high. The settling is better in Figure 10a than in Figure 10b. The wastewater is still very turbid, such that turbidity is not measurable.

From the experimental results, the best pharmaceutical system is as follows: sequential dosing, a pharmaceutical action time of 2 min, a stirring intensity of 500 r/min, a CaO dosage of 200 g/m^3^, a PAC dosage of 5 g/m^3^, and a PAM-New pharmaceutical dosage of 12 g/m^3^. The turbidity of the treated wastewater was 97.30 NTU, which met the industrial wastewater discharge standard.

### 3.2. Zeta Potential Measurement

#### 3.2.1. Sediment Surface Zeta Potential Detection

**Figure 11:**
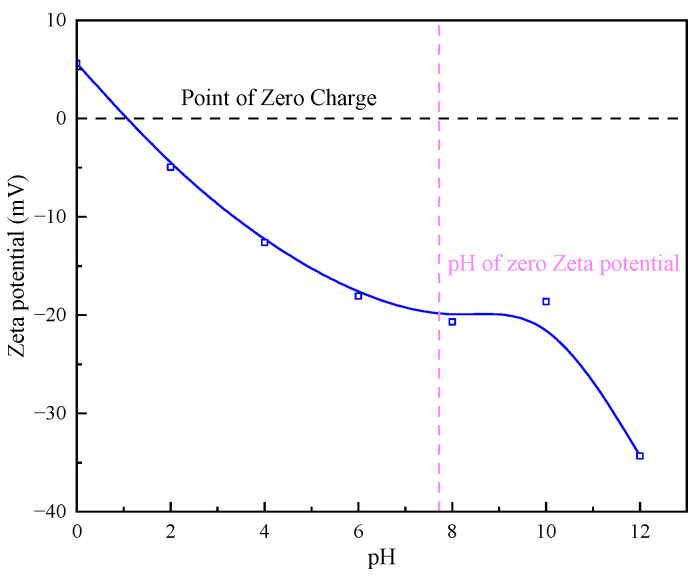
Effect of different pH on zeta potential of particle surface.

It is evident from Figure 11 that the zero electric point of the particles is between pH 1 and 2, as the quarry particles are composed of a variety of minerals. At pH 12, the absolute value of the zeta potential is the largest, i.e., 34.32. At pH 2, the absolute value of the zeta potential is the smallest, i.e., 4.96. It indicates that, when the pH is larger, the electrostatic repulsive force between the particles is large, and adsorption does not occur easily. Therefore, controlling the pH value of the solution can reduce the influence of electrostatic repulsion, which is conducive to interparticle adsorption and sedimentation.

#### 3.2.2. Effect of Single Flocculant Dosage on Zeta Point Position of Quarry Wastewater Particles

**Figure 12:**
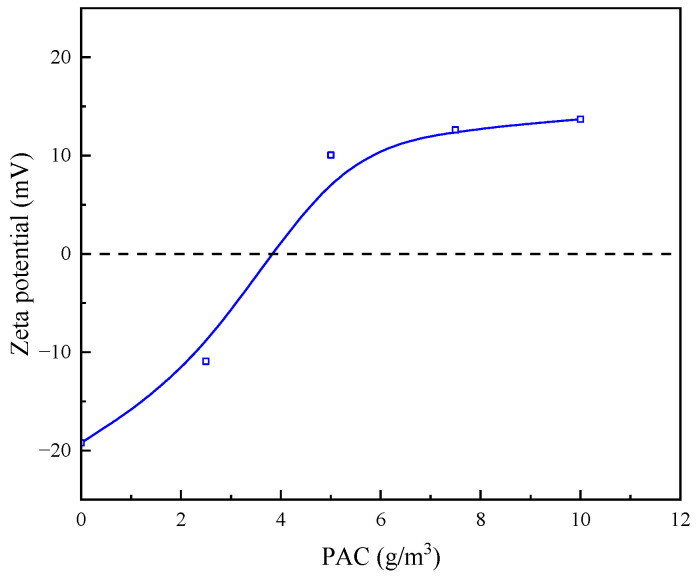
Effect of same concentration of PAC on zeta potential of particle surface.

It is evident from Figure 12 that, with the increase in PAC concentration, the zeta potential on the surface of the mineral particles shifted to the positive direction, as PAC is a cationic flocculant, which causes the neutralisation of the particle surface charge; with the increase in PAC concentration, the charging effect is continuously strengthened, and the effect of flocculation and sedimentation is enhanced. Combined with the experimental results, it shows that PAC achieves the flocculation and sedimentation effect through the electrical neutralisation of the particle surface.

**Figure 13:**
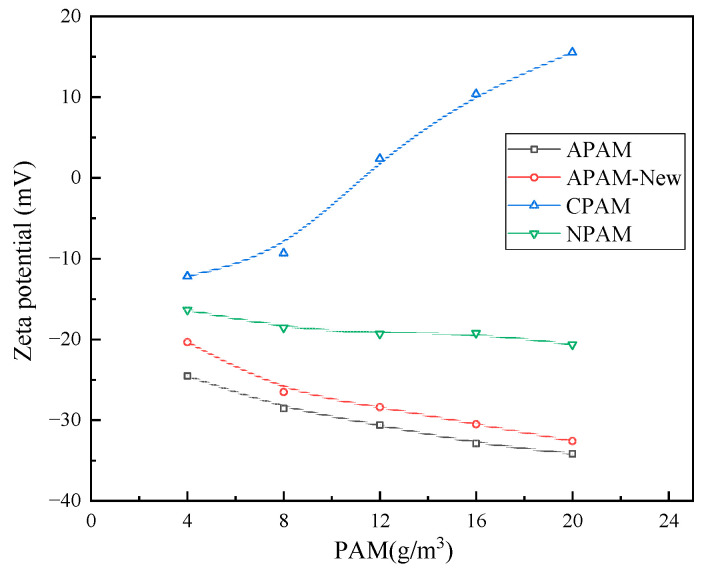
Effect of different ionic PAM on zeta potential of particle surface zeta at different concentrations.

It is evident from Figure 13 that the effect of different ionic PAM on the surface potential of the quarry particles varied, as the zeta potential shifted in a positive direction after the action of CPAM. This occurs because CPAM is a cationic flocculant, which neutralises the surface charge of the particles, and the value of the potential gradually becomes smaller. When the concentration is increased to 16 g/m^3^, the potential remains unchanged, indicating that the saturation of the charge neutralisation ability has occurred at this time. When APAM and NPAM are used in the same concentration, the change in zeta potential after the action of ionic APAM is not large, indicating that the electric neutralisation effect is only a part of the APAM flocculation force.

When a 12 g/m^3^ concentration of APAM is used, the best flocculation effect is observed among the three types of PAM. Furthermore, based on the experimental results, this is the best concentration of PAM.

**Figure 14:**
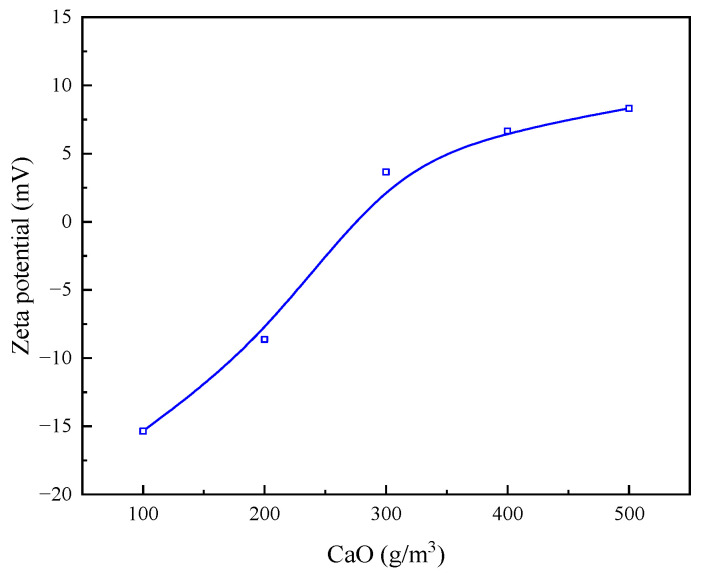
Effect of different concentrations of CaO on zeta potential of particle surface.

It is evident from Figure 14 that, with the increase in the concentration of CaO, the zeta potential of the surface of the mineral particles showed a certain magnitude of positive shift, which is due to the fact that Ca^2+^ compresses the double-electron layer mainly through electrostatic adsorption and hydroxyl complexation, resulting in the absolute value of the kinetic potential on the surface of the particles decreasing with the increase in the concentration. Based on the experimental flocculation effect, potential change, and cost, the flocculation effect of 200 g/m^3^ CaO can meet the requirements.

#### 3.2.3. Effect of Optimum Flocculating Agent Dosage on Zeta Potential of Quarry Wastewater Particles

The observed absolute zeta potential values (Table 3) in descending order are as follows: APAM > APAM-New > PAC > CaO > CaO + PAC + APAM > CaO + PAC + APAM-New. Notably, the CaO + PAC + APAM-New formulation induced the most pronounced alteration, reducing the surface zeta potential to 2.66 mV. This result indicates its superior electroneutralisation effect and strong adsorption capacity toward mineral particles, whereas conventional APAM exhibited minimal influence on surface potential. The integrated analysis of flocculation efficacy and zeta potential variations revealed that the optimal dosage of CaO + PAC + APAM-New achieved the most effective flocculation performance, with the treated supernatant exhibiting the highest transparency and lowest turbidity.

### 3.3. Optical Microscope Analysis

**Figure 15:**
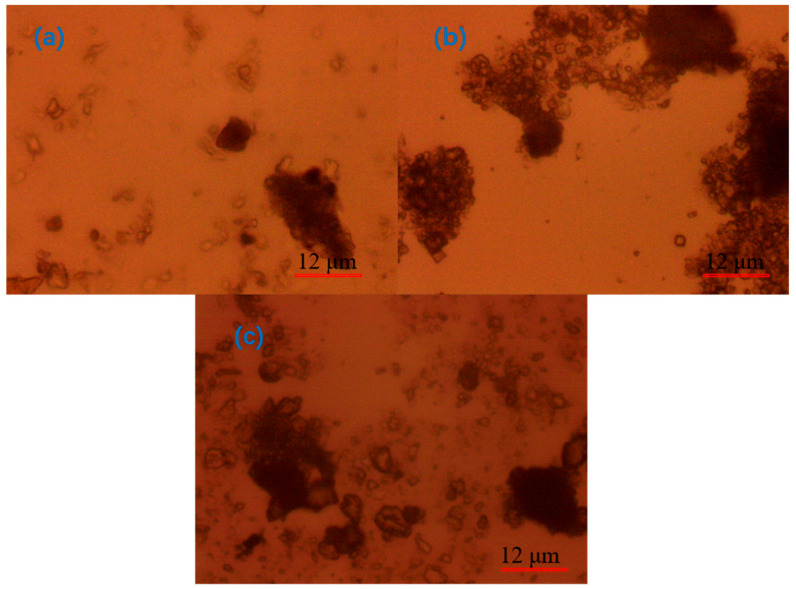
(**a**) Floc in its natural state; (**b**) micromorphology of floc after using CaO as flocculant; (**c**) PAC.

As illustrated in Figure 15, the microscopic morphology of the flocs in different states of the quarry particles is observed under the 640-fold lens. In the natural state (Figure 15a), the quarry particles exhibit increased dispersion, an absence of discernible connections between particles, a more uniform distribution, and clumps between the quarry particles that are more loosely packed, with a smaller clump size and a uniform distribution.

Figure 15b illustrates the effect of adding the regulator CaO. There are obvious connections between the quarry particles; the particles are agglomerated, and there is no obvious particle distribution around the flocs, which indicates that the flocculation and clarification effect is adequate; however, the connection between the particles is not tight, which indicates that the flocs are not stable. Figure 15c shows the effect of the addition of the inorganic flocculant PAC. The connections between the quarry particles are clearly evident, but the agglomeration is reduced, and the connection between the particles is not tight.

**Figure 16:**
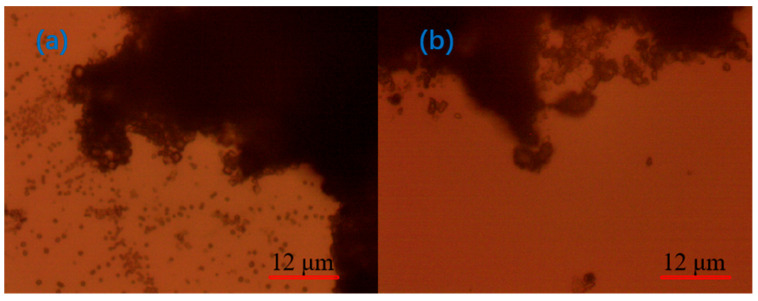
Micromorphology of flocs after addition of (**a**) APAM and (**b**) APAM-New.

Figure 16 shows the microscopic morphology of flocs after adding different APAM. After adding the anionic organic flocculant PAM, it was observed that the connection between particles was tight and firm, and flocs were larger and stable, but there were a large number of unattached tiny particles around the flocs, which indicated that the clarification effect of the anionic organic flocculant was poor. After adding APAM-New, the flocs were more closely connected to each other, and there were fewer unattached particles near the flocs than those of APAM, which indicated that the flocculation and clumping effect of APAM-New was more stable than that of APAM.

The above analysis found that the addition of a single agent could not achieve the tight connection of flocs, and there was no obvious distribution of unconnected particles around them. This is the same as the experimental results, and to improve the flocculation effect, a combination of flocculants needs to be tested.

**Figure 17:**
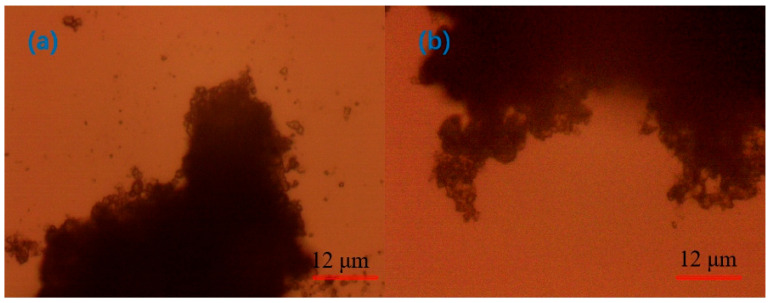
Flocculation micromorphology after addition of (**a**) CaO + PAC + APAM and (**b**) CaO + PAC + APAM-New.

It is evident from Figure 17, which shows the microscopic morphology of flocs after the addition of different agent combinations, that, after adding different optimal agent combinations to the quarry wastewater, the connection between the quarry particles was tight, and the flocs were large and thick under the microscope, which indicated that the floc combination was more stable; however, the flocs were more compact and stable after the addition of CaO + PAC + APAM-New than CaO + PAC + APAM, which indicated that the CaO + PAC + APAM-New exhibited a better flocculation effect. Moreover, this flocculation effect was better than that observed by adding a single flocculant. This observation is consistent with the experimental results.

### 3.4. Theoretical Analysis of DLVO of Wastewater Particles


(1) $$V_{T}=V_{A}+V_{R}$$

*V_T_* denotes the total energy between flocculated charged colloidal particles. *V_A_* denotes the van der Waals energy, and *V_R_* denotes the electrostatic energy.

(2) $$V_{A}=-\frac{AR}{6H}$$

**Figure 18:**
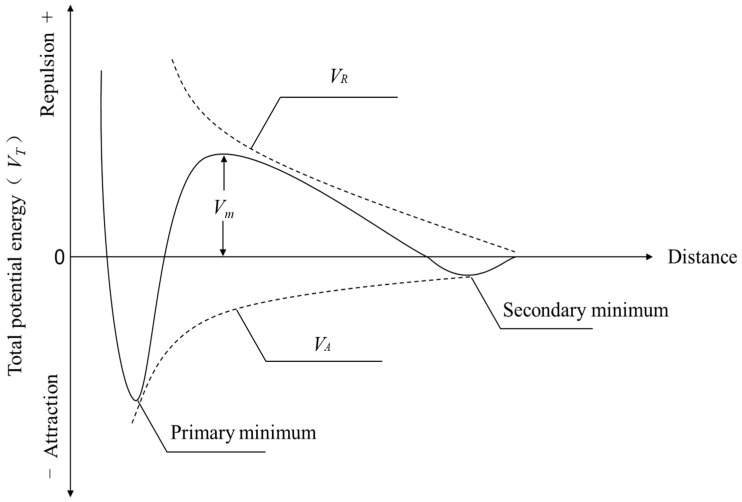
Schematic diagram of theoretical total potential energy curve of DLVO.

The effective Hamaker constant, *A*, is expressed in units of J. *R* represents the radius of the colloidal particles, and *H* represents the distance between the particles.

(3) $$V_{R}=2\pi \varepsilon R\varphi_{0}^{2}\ln(1-e^{-2KH})$$

(4) $$\varepsilon =\varepsilon_{0}\varepsilon_{r}$$

where *ε*_0_ denotes the vacuum absolute dielectric constant in F/m, *ε_r_* denotes the relative permittivity, *φ*_0_ denotes the particle surface potential, *e* denotes the electron charge, and *k* denotes the reciprocal of the Debye length. The Schematic diagram of theoretical total potential energy curve of DLVO is shown in Figure 18.

The microparticles in the slurry used in this study are spherical, and the distance between particles is the distance between the two spheres. The average particle size of the particles in the slurry was 7.52 μm.

Then, the Hamaker constant for the interaction of the tiny particles with the aqueous medium is as follows:

(5) $$A\approx {\left(\sqrt{A_{1}}-\sqrt{A_{2}}\right)}^{2}$$

Based on reference [33], the Hamaker constant for *A*_1_ is 1.2 × 10^−19^ J for the tiny particles themselves, and *A*_2_ is 4.84 × 10^−20^ J for water.

Based on reference [33], the vacuum absolute permittivity of tiny particles is *ε*_0_ = 8.854 × 10^−12^ F/m, and the permittivity of water is *ε_r_* = 78.5. Based on Equations (3) and (4), the permittivity of tiny particles in water is as follows:

(6) $$\varepsilon =\varepsilon_{0}\varepsilon_{r}=8.854\times 10^{-12}\;F/m\times 78.5\;=6.95039\times 10^{-14}\;F/m$$

Based on reference [34], the inverse k of Debye length is 0.180 nm^−1^, and the zeta potential is given in Section 1. The temperature condition of this experiment is room temperature, i.e., 25 °C. Combined with the theoretical Equations of DLVO without the addition of agents, the particle spacing is taken as 1^−30^ nm for the calculation, and the process of potential energy calculation is as follows:

The interparticle distance for *H* is 1 nm:

(7) $$V_{A}=-\frac{AR}{6H}=-\frac{1.592\times 10^{-20}\times 7.52\times 10^{-6}}{12\times 10^{-9}}=-9.97\times 10^{-18}J$$

(8) $$\begin{array}{l} V_{R}=2\pi \varepsilon R\varphi_{0}^{2}\ln(1-e^{-2KH})=2\times \pi \times 6.95039\times 7.52\times (-19{)}^{2}\times \ln(1-e^{-2\times 0.180\times H})\times 10^{-20}\\ =14.82\times 10^{-18}J \end{array}$$

(9) $$V_{T}=V_{A}+V_{R}=(14.82-9.97)\times 10^{-18}=4.85\times 10^{-18}J$$

The interparticle distance for *H* is 2 nm:

(10) $$V_{A}=-\frac{AR}{6H}=-\frac{1.592\times 10^{-20}\times 7.52\times 10^{-6}}{12\times 10^{-9}\times 2}=-4.985\times 10^{-18}J$$

(11) $$\begin{array}{l} V_{R}=2\pi \varepsilon R\varphi_{0}^{2}\ln(1-e^{-2KH})=2\times 3.14\times 6.95039\times 7.52\times (-19{)}^{2}\times \ln(1-e^{-2\times 0.180\times H})\times 10^{-20}\\ =12.82\times 10^{-18}J \end{array}$$

(12) $$V_{T}=V_{A}+V_{R}=(12.82-4.985)\times 10^{-18}=7.835\times 10^{-18}J$$

The interparticle distance for *H* is 3 nm:

(13) $$V_{A}=-\frac{AR}{6H}=-\frac{1.592\times 10^{-20}\times 7.52\times 10^{-6}}{12\times 10^{-9}\times 3}=-3.325\times 10^{-18}J$$

(14) $$\begin{array}{l} V_{R}=2\pi \varepsilon R\varphi_{0}^{2}\ln(1-e^{-2KH})=2\times 3.14\times 6.95039\times 7.52\times (-19{)}^{2}\times \ln(1-e^{-2\times 0.180\times H})\times 10^{-20}\\ =10.06\times 10^{-18}J \end{array}$$

(15) $$V_{T}=V_{A}+V_{R}=(10.06-3.325)\times 10^{-18}=6.735\times 10^{-18}J$$

The equations are similarly derived up to the interparticle distance for *H* of 30 nm.

**Figure 19:**
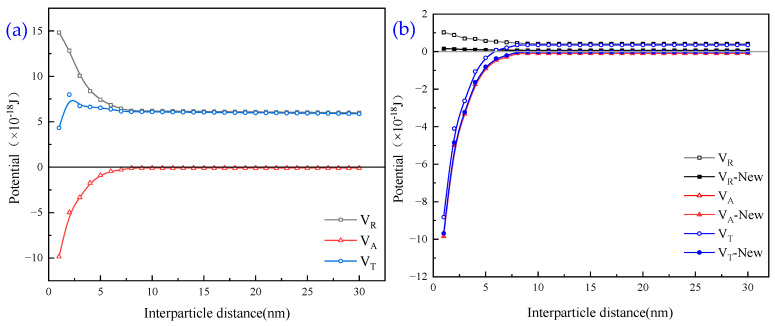
(**a**) Total potential energy between tiny particles; (**b**) potential energy changes between APAM and APAM-new combinations.

The variation in interparticle interaction potential energy versus interparticle distance is presented in Figure 19, where *V_T_* is the total energy between flocculated charged colloidal particles, *V_A_* is the van der Waals interaction energy, and *V_R_* is the electrostatic interaction energy.

It is evident from Figure 19a that, in the case of no flocculant addition, the total potential energy between particles first increases with the distance between particles and then tends to stabilise, and the overall electrostatic force (*V_R_*) is positive; for the repulsive potential energy, with the increase in the distance, the absolute value of the *V_R_* gradually decreases. The van der Waals force (*V_A_*) is negative; for the attraction of the potential energy, with the increase in the distance, the absolute value of the *V_A_* gradually decreases. The total potential energy (*V_T_*) fluctuates, which is due to the residual flocculating agent that brings particles closer to each other. There is little adsorption. As the distance between the particles increases, the total potential energy tends to stabilise, indicating that the particles at a distance are more stable and do not form clusters.

It is evident from Figure 19b that the potential energy between particles under the best flocculating agent combination is shifted compared to that without flocculating agent addition. With the addition of flocculating agent dosage, the electrostatic repulsive force (*V_R_*) decreases greatly; the absolute value of van der Waals’ force (*V_T_*) increases; therefore, the total potential energy (*V_T_*) is negative overall, and at this time, the interparticle force is attraction, and the particles can spontaneously coalesce to form a larger agglomeration, with a good flocculating effect.

**Figure 20:**
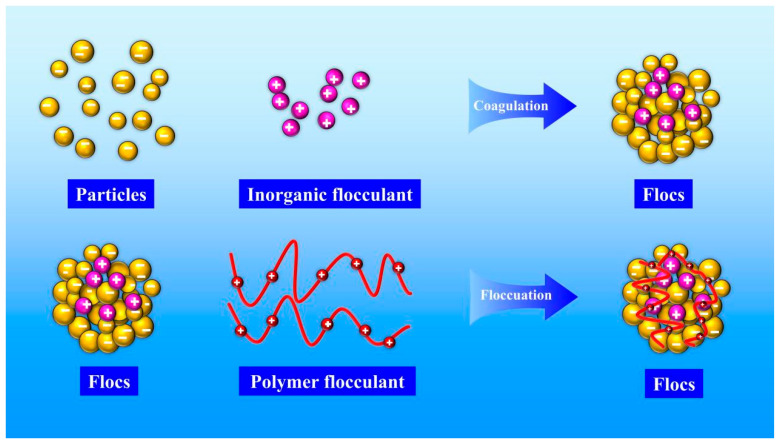
Flocculation mechanism.

During the flocculation process, negatively charged primary particles are destabilised by the addition of positively charged inorganic coagulants, forming loosely aggregated flocs. The subsequent introduction of positively charged organic flocculants enhances floc stability through bridging. The simulation model of the flocculation process is presented in Figure 20.

## 4. Conclusions

The main conclusions drawn from this study are as follows:

(1) When exploring the effect of flocculants added individually on the flocculation effect of quarry wastewater, the regulator agent has the best effect on water quality improvement, followed by inorganic flocculants, and organic flocculants have the worst effect on water quality improvement. Organic flocculants have the fastest flocculation and settling rate, followed by inorganic flocculants, and the regulator agent has the slowest settling rate. Thus, we selected inorganic flocculants with the best combined effect, i.e., PAC and PAM, and the regulator agent CaO as the flocculants for the subsequent experiments.
(2) In the combination experiment of APAM and PAC, the particle settling rate is accelerated, and the upper layer of the clear liquid is more turbid. In the combination experiment of APAM and CaO, the particle settling rate is enhanced to a lesser degree, and the upper layer of the clear liquid is clearer. The combination experiment of CaO, PAM, and PAC found that the particle settling rate is faster, and the upper layer of the clear liquid is clearer, but it still fails to meet the industry standards. Therefore, combined with the actual needs of the site, under the same conditions as those used for CaO and PAC, the settling effect of the PAM-modified agent is better than that of PAM. Therefore, the best results of the experiments are obtained under the following conditions: sequential dosing, an agent action time of 2 min, a stirring intensity of 500 r/min, a CaO dosage of 200 g/m^3^, a PAC dosage of 5 g/m^3^, and a PAM-modified agent amount of 12 g/m^3^. Under these conditions, the wastewater turbidity was 97.30 NTU.
(3) By detecting the zeta potential on the surface of settled particles, it was found that the zeta potential on the surface of the particles increased with the increase in the concentration of the inorganic flocculant PAC and regulator agent CaO, which indicated that PAC and CaO mainly promoted mutual flocculation and precipitation among particles through the effect of electroneutralisation. The zeta potential on the surface of the particles did not change much with the increase in the concentration of the organic flocculant, which indicated that the inorganic flocculant mainly promoted mutual adsorption and precipitation among particles through adsorption and bridging. This suggests that the inorganic flocculant mainly promotes mutual adsorption and precipitation between particles through adsorption and bridging.
(4) Under the optical microscope, it was observed that, after adding CaO to the quarry wastewater, the flocs formed by tiny particles were obvious but not compact; after adding PAC, the flocs formed by tiny particles were smaller; after adding PAM, the flocs were larger but compact; and after the optimal combination of agents was added, the flocs were stable, and the agglomerates were large.
(5) The theoretical analysis of DLVO shows that the total potential energy between particles in the quarry wastewater in the natural state is positive, and there is a large repulsive force between these particles, which results in turbid wastewater. After adding the optimal combination of agents, the total potential energy between particles exhibits a negative value, indicating that the particles begin to attract each other and form larger flocs after the addition.

## Figures and Tables

**Table 1:** Elemental composition of mineral particles (wt%).

Element	Content	Element	Content
O	47.047	Mn	0.285
Si	28.212	P	0.088
Al	10.723	S	0.048
Fe	4.33	Cl	0.029
K	3.453	Zr	0.025
Ca	3.33	Sr	0.023
Na	1.233	Rb	0.021
Mg	0.585	Zn	0.013
Ti	0.55	Ga	0.003

**Table 2:** pH values of aqueous waste solution after adding CaO.

CaO (g/m^3^)	0	100	200	300	400	500
pH	7.96	8.96	9.91	10.48	10.88	11.21

**Table 3:** Influence of optimal reagent dosages on zeta potential of particle surfaces.

Types of Flocculation Chemicals	Blank Test	CaO	PAC	APAM	APAM-New	APAM Combinations	APAM-New Combinations
Zeta potential (mV)	−19.83	−8.63	−10.94	−30.65	−28.42	−5.35	−2.66

## Data Availability

The original contributions presented in this study are included in the article. Further inquiries can be directed to the corresponding authors.
